# 1′,5-Dinitro-2′-phenyl-2′,3′,5′,6′,7′,7a’-hexa­hydro­spiro­[indoline-3,3′-1′*H*-pyrrolizin]-2-one

**DOI:** 10.1107/S1600536808025038

**Published:** 2008-08-13

**Authors:** Yaghoub Sarrafi, Kamal Alimohammadi

**Affiliations:** a Department of Chemistry, University of Mazandaran, 47415 Babolsar, Iran

## Abstract

In the title cyclo­adduct, C_20_H_18_N_4_O_5_, the rings of the pyrrolizine system adopt envelope conformations. A centrosymmetric dimer is formed *via* inter­molecular N—H⋯O hydrogen bonds between the indolinone rings.

## Related literature

For related literature, see: De March *et al.* (2002 ▶); Fejes *et al.* (2001 ▶); Karthikeyan *et al.* (2007 ▶); Usha *et al.* (2005*a* ▶,*b* ▶); Liddell (1998 ▶); Michael (1997 ▶).
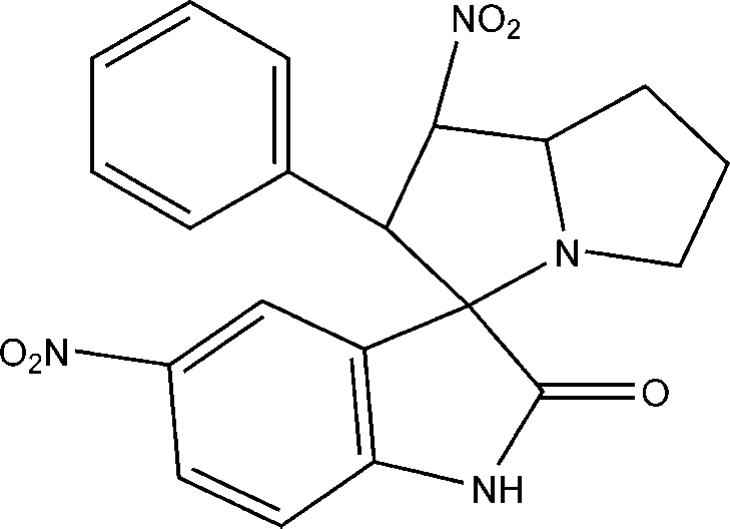


## Experimental

### 

#### Crystal data

C_20_H_18_N_4_O_5_
*M* *_r_* = 394.38
Monoclinic, 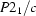

*a* = 13.998 (4) Å
*b* = 7.963 (3) Å
*c* = 16.359 (6) Å
β = 99.695 (11)°
*V* = 1797.5 (10) Å^3^
*Z* = 4
Mo *K*α radiation
μ = 0.11 mm^−1^
*T* = 120 (2) K
0.26 × 0.18 × 0.12 mm

#### Data collection

Bruker SMART 1000 CCD area-detector diffractometer
Absorption correction: none
13372 measured reflections
4316 independent reflections
2227 reflections with *I* > 2σ(*I*)
*R*_int_ = 0.044

#### Refinement

*R*[*F* ^2^ > 2σ(*F* ^2^)] = 0.057
*wR*(*F* ^2^) = 0.114
*S* = 1.00
4316 reflections
262 parameters
H-atom parameters constrained
Δρ_max_ = 0.31 e Å^−3^
Δρ_min_ = −0.25 e Å^−3^

### 

Data collection: *SMART* (Bruker, 2007 ▶); cell refinement: *SAINT* (Bruker, 2007 ▶); data reduction: *SAINT*; program(s) used to solve structure: *SHELXTL* (Sheldrick, 2008 ▶); program(s) used to refine structure: *SHELXTL*; molecular graphics: *SHELXTL*; software used to prepare material for publication: *SHELXTL*.

## Supplementary Material

Crystal structure: contains datablocks I, global. DOI: 10.1107/S1600536808025038/om2253sup1.cif

Structure factors: contains datablocks I. DOI: 10.1107/S1600536808025038/om2253Isup2.hkl

Additional supplementary materials:  crystallographic information; 3D view; checkCIF report

## Figures and Tables

**Table 1:** Hydrogen-bond geometry (Å, °)

*D*—H⋯*A*	*D*—H	H⋯*A*	*D*⋯*A*	*D*—H⋯*A*
N1—H1*A*⋯O1^i^	0.86	1.97	2.808 (2)	164

Symmetry code: (i) 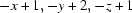.

**Fig. 1.**
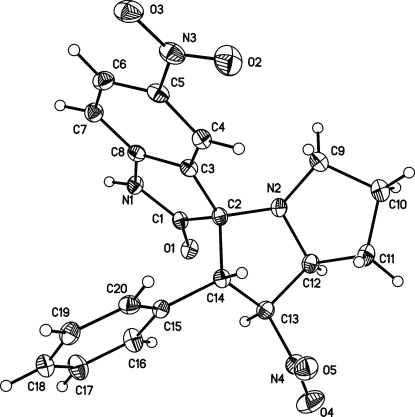
The molecular structure of the title compound with the numbering scheme for the atoms and 30% probability displacement ellipsoids.

**Fig. 2.**
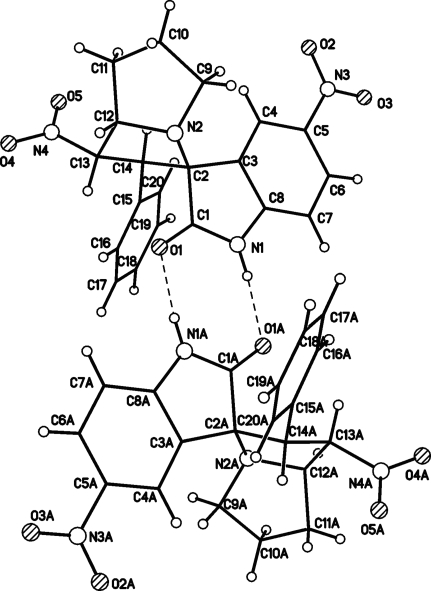
H-bonding dimer diagram of the molecules with hydrogen bonds shown as dashed lines.

## supplementary crystallographic information

### Comment

1,3-Dipolar cycloadditions form a subject of intensive research in organic synthesis in view of their great synthetic potential (Karthikeyan *et al.*, 2007). Azomethine ylides are reactive and versatile 1,3-dipoles, which readily react with diverse dipolarophiles affording pyrrolizines, pyrrolidines and pyrazolidines (Fejes *et al.*, 2001; De March *et al.*, 2002). The pyrrolizine substructure occurs in many natural products of potential use in medicine and agriculture (Liddell, 1998; Michael, 1997). The title compound was synthesized by the multicomponent 1,3-dipolar cycloaddition of azomethine ylide, derived from 5-nitroisatin and proline by a decarboxylative route, and *trans*-β-nitrostyrene. The geometry of the crystal structure (Fig. 1) is similar to reported compounds (Usha *et al.*, 2005a, 2005b). The molecular structure of the title compound shows a centrosymmetric dimer (Fig. 2) *via* N—H···O intermolecular interactions and crystal packing is stabilized through intermolecular C—H···O and C—H···π interactions.

### Refinement

All hydrogen atoms were refined in isotropic approximation in riding model with the *U*_iso_(H) parameters equal to 1.2 *U*_eq_(Ci) where U(Ci) are the equivalent thermal parameters of the atoms to which the corresponding H atoms are bonded and C-H = 0.93-0.98 Å.

### Crystal data

**Table:**

C_20_H_18_N_4_O_5_	*F*_000_ = 824
*M**_r_* = 394.38	*D*_x_ = 1.457 Mg m^−^^3^
Monoclinic, *P*2_1_/*c*	Mo *K*α radiation λ = 0.71073 Å
Hall symbol: -P 2ybc	Cell parameters from 438 reflections
*a* = 13.998 (4) Å	θ = 2.1–19.3º
*b* = 7.963 (3) Å	µ = 0.11 mm^−^^1^
*c* = 16.359 (6) Å	*T* = 120 (2) K
β = 99.695 (11)º	Prism, yellow
*V* = 1797.5 (10) Å^3^	0.26 × 0.18 × 0.12 mm
*Z* = 4	

### Data collection

**Table:**

Bruker SMART 1000 CCD area-detector diffractometer	2227 reflections with *I* > 2σ(*I*)
Radiation source: fine-focus sealed tube	*R*_int_ = 0.044
Monochromator: graphite	θ_max_ = 28.0º
*T* = 120(2) K	θ_min_ = 2.5º
φ and ω scans	*h* = −17→18
Absorption correction: none	*k* = −10→10
13372 measured reflections	*l* = −21→21
4316 independent reflections	

### Refinement

**Table:**

Refinement on *F*^2^	Secondary atom site location: difference Fourier map
Least-squares matrix: full	Hydrogen site location: difference Fourier map
*R*[*F*^2^ > 2σ(*F*^2^)] = 0.057	H-atom parameters constrained
*wR*(*F*^2^) = 0.114	*w* = 1/[σ^2^(*F*_o_^2^) + (0.02*P*)^2^ + 1.3*P*] where *P* = (*F*_o_^2^ + 2*F*_c_^2^)/3
*S* = 1.00	(Δ/σ)_max_ < 0.001
4316 reflections	Δρ_max_ = 0.31 e Å^−^^3^
262 parameters	Δρ_min_ = −0.25 e Å^−^^3^
Primary atom site location: structure-invariant direct methods	Extinction correction: none

### Special details

**Table:**

Experimental. A mixture of 5-nitroisatin (0.192 g, 1 mmol), proline (0.115 g, 1 mmol), and *trans*-β-nitrostyrene (0.149 g, 1 mmol) in ethanol (10 ml) was stirred at refluxed for 1 h. After completion of the reaction, as indicated by TLC, to solution was added water (25 ml) and the precipitated solid was separated by filtration. The pure cycloadduct was obtained by recrystallization from ethanol.
Geometry. All e.s.d.'s (except the e.s.d. in the dihedral angle between two l.s. planes) are estimated using the full covariance matrix. The cell e.s.d.'s are taken into account individually in the estimation of e.s.d.'s in distances, angles and torsion angles; correlations between e.s.d.'s in cell parameters are only used when they are defined by crystal symmetry. An approximate (isotropic) treatment of cell e.s.d.'s is used for estimating e.s.d.'s involving l.s. planes.
Refinement. Refinement of *F*^2^ against ALL reflections. The weighted *R*-factor *wR* and goodness of fit *S* are based on *F*^2^, conventional *R*-factors *R* are based on *F*, with *F* set to zero for negative *F*^2^. The threshold expression of *F*^2^ > σ(*F*^2^) is used only for calculating *R*-factors(gt) *etc*. and is not relevant to the choice of reflections for refinement. *R*-factors based on *F*^2^ are statistically about twice as large as those based on *F*, and *R*- factors based on ALL data will be even larger.

### Fractional atomic coordinates and isotropic or equivalent isotropic displacement parameters (Å^2^)

**Table:**

	*x*	*y*	*z*	*U*_iso_*/*U*_eq_	
O1	0.42737 (11)	0.8306 (2)	0.53311 (9)	0.0310 (4)	
O2	−0.02802 (13)	1.0541 (3)	0.22715 (12)	0.0518 (5)	
O3	0.03964 (13)	1.2271 (2)	0.15286 (11)	0.0449 (5)	
O4	0.27784 (16)	0.2753 (2)	0.54074 (12)	0.0596 (6)	
O5	0.17142 (13)	0.3390 (2)	0.43381 (11)	0.0441 (5)	
N1	0.39099 (13)	1.0143 (2)	0.42331 (11)	0.0271 (5)	
H1A	0.4465	1.0625	0.4266	0.033*	
N2	0.21896 (13)	0.8303 (2)	0.52112 (11)	0.0276 (5)	
N3	0.04377 (16)	1.1279 (3)	0.21107 (13)	0.0366 (5)	
N4	0.23870 (16)	0.3752 (3)	0.48895 (13)	0.0360 (5)	
C1	0.37153 (16)	0.8895 (3)	0.47458 (14)	0.0260 (5)	
C2	0.26499 (15)	0.8336 (3)	0.44673 (13)	0.0240 (5)	
C3	0.23048 (16)	0.9555 (3)	0.37701 (13)	0.0247 (5)	
C4	0.14310 (17)	0.9760 (3)	0.32541 (13)	0.0277 (5)	
H4A	0.0896	0.9110	0.3315	0.033*	
C5	0.13764 (17)	1.0973 (3)	0.26379 (14)	0.0283 (6)	
C6	0.21565 (18)	1.1911 (3)	0.24975 (15)	0.0325 (6)	
H6A	0.2090	1.2681	0.2064	0.039*	
C7	0.30444 (17)	1.1698 (3)	0.30081 (14)	0.0304 (6)	
H7A	0.3586	1.2309	0.2924	0.036*	
C8	0.30976 (16)	1.0547 (3)	0.36449 (13)	0.0244 (5)	
C9	0.12231 (17)	0.9058 (3)	0.52006 (15)	0.0346 (6)	
H9A	0.0875	0.9154	0.4638	0.042*	
H9B	0.1279	1.0164	0.5453	0.042*	
C10	0.07094 (18)	0.7847 (3)	0.57053 (16)	0.0373 (6)	
H10A	0.0881	0.8064	0.6295	0.045*	
H10B	0.0011	0.7911	0.5544	0.045*	
C11	0.10985 (18)	0.6159 (3)	0.54746 (17)	0.0383 (6)	
H11A	0.0999	0.5293	0.5868	0.046*	
H11B	0.0801	0.5814	0.4922	0.046*	
C12	0.21678 (17)	0.6552 (3)	0.55171 (14)	0.0296 (6)	
H12A	0.2497	0.6497	0.6095	0.036*	
C13	0.27408 (17)	0.5532 (3)	0.49667 (14)	0.0297 (6)	
H13A	0.3429	0.5540	0.5215	0.036*	
C14	0.26082 (16)	0.6474 (3)	0.41508 (13)	0.0256 (5)	
H14A	0.1945	0.6264	0.3867	0.031*	
C15	0.32836 (17)	0.6039 (3)	0.35484 (14)	0.0272 (5)	
C16	0.42558 (18)	0.5628 (3)	0.38008 (15)	0.0371 (6)	
H16A	0.4513	0.5618	0.4363	0.044*	
C17	0.48456 (19)	0.5237 (3)	0.32300 (16)	0.0417 (7)	
H17A	0.5493	0.4969	0.3411	0.050*	
C18	0.44760 (19)	0.5243 (3)	0.23930 (15)	0.0387 (6)	
H18A	0.4870	0.4960	0.2010	0.046*	
C19	0.35159 (19)	0.5672 (3)	0.21255 (15)	0.0359 (6)	
H19A	0.3266	0.5702	0.1562	0.043*	
C20	0.29304 (18)	0.6057 (3)	0.27026 (14)	0.0311 (6)	
H20A	0.2285	0.6335	0.2519	0.037*	

### Atomic displacement parameters (Å^2^)

**Table:**

	*U*^11^	*U*^22^	*U*^33^	*U*^12^	*U*^13^	*U*^23^
O1	0.0260 (9)	0.0382 (10)	0.0268 (9)	−0.0038 (8)	−0.0010 (7)	0.0041 (8)
O2	0.0293 (11)	0.0701 (15)	0.0526 (12)	0.0004 (10)	−0.0030 (9)	0.0051 (10)
O3	0.0505 (12)	0.0470 (11)	0.0340 (11)	0.0179 (10)	−0.0023 (9)	0.0041 (9)
O4	0.0802 (16)	0.0409 (12)	0.0554 (13)	0.0026 (11)	0.0053 (11)	0.0165 (10)
O5	0.0453 (12)	0.0390 (11)	0.0477 (12)	−0.0096 (9)	0.0067 (10)	−0.0070 (9)
N1	0.0195 (10)	0.0318 (11)	0.0292 (10)	−0.0042 (9)	0.0018 (8)	0.0027 (9)
N2	0.0246 (11)	0.0306 (11)	0.0281 (11)	−0.0027 (9)	0.0060 (8)	−0.0001 (9)
N3	0.0357 (13)	0.0423 (13)	0.0299 (12)	0.0116 (11)	−0.0001 (10)	−0.0051 (10)
N4	0.0405 (13)	0.0319 (12)	0.0369 (13)	−0.0016 (11)	0.0105 (11)	0.0010 (10)
C1	0.0254 (13)	0.0306 (13)	0.0226 (12)	0.0000 (11)	0.0056 (10)	−0.0012 (10)
C2	0.0192 (12)	0.0285 (13)	0.0243 (12)	−0.0016 (10)	0.0037 (9)	−0.0010 (10)
C3	0.0234 (12)	0.0260 (12)	0.0246 (12)	0.0010 (10)	0.0033 (10)	−0.0023 (10)
C4	0.0261 (13)	0.0287 (13)	0.0282 (13)	−0.0024 (11)	0.0039 (10)	−0.0048 (10)
C5	0.0254 (13)	0.0328 (13)	0.0243 (12)	0.0068 (11)	−0.0023 (10)	−0.0042 (10)
C6	0.0379 (15)	0.0292 (14)	0.0294 (13)	0.0040 (12)	0.0028 (11)	0.0026 (11)
C7	0.0283 (13)	0.0313 (13)	0.0315 (13)	−0.0008 (11)	0.0051 (11)	0.0013 (11)
C8	0.0239 (13)	0.0244 (12)	0.0241 (12)	0.0011 (10)	0.0016 (10)	−0.0040 (10)
C9	0.0328 (14)	0.0361 (15)	0.0370 (15)	0.0037 (12)	0.0116 (12)	−0.0014 (12)
C10	0.0324 (14)	0.0451 (16)	0.0360 (15)	0.0002 (13)	0.0099 (12)	−0.0006 (12)
C11	0.0349 (15)	0.0368 (15)	0.0457 (16)	−0.0088 (12)	0.0141 (12)	0.0021 (12)
C12	0.0322 (14)	0.0326 (14)	0.0243 (12)	−0.0009 (11)	0.0057 (10)	0.0022 (10)
C13	0.0281 (13)	0.0275 (13)	0.0340 (14)	−0.0035 (11)	0.0062 (11)	−0.0013 (11)
C14	0.0227 (12)	0.0287 (13)	0.0250 (12)	−0.0018 (10)	0.0029 (10)	−0.0012 (10)
C15	0.0263 (13)	0.0256 (12)	0.0304 (13)	−0.0023 (11)	0.0072 (10)	0.0010 (10)
C16	0.0297 (14)	0.0531 (17)	0.0275 (13)	0.0015 (13)	0.0021 (11)	−0.0005 (12)
C17	0.0239 (14)	0.0599 (19)	0.0413 (16)	0.0031 (13)	0.0054 (12)	−0.0014 (14)
C18	0.0427 (17)	0.0420 (16)	0.0351 (15)	0.0024 (13)	0.0171 (13)	0.0005 (12)
C19	0.0437 (16)	0.0397 (15)	0.0241 (13)	0.0025 (13)	0.0049 (12)	0.0081 (11)
C20	0.0317 (14)	0.0287 (13)	0.0316 (14)	0.0051 (11)	0.0018 (11)	0.0029 (11)

### Geometric parameters (Å, °)

**Table:**

O1—C1	1.223 (2)	C9—H9A	0.9700
O2—N3	1.230 (3)	C9—H9B	0.9700
O3—N3	1.232 (3)	C10—C11	1.521 (3)
O4—N4	1.223 (3)	C10—H10A	0.9700
O5—N4	1.224 (2)	C10—H10B	0.9700
N1—C1	1.357 (3)	C11—C12	1.519 (3)
N1—C8	1.398 (3)	C11—H11A	0.9700
N1—H1A	0.8600	C11—H11B	0.9700
N2—C2	1.470 (3)	C12—C13	1.535 (3)
N2—C9	1.478 (3)	C12—H12A	0.9800
N2—C12	1.483 (3)	C13—C14	1.515 (3)
N3—C5	1.466 (3)	C13—H13A	0.9800
N4—C13	1.500 (3)	C14—C15	1.516 (3)
C1—C2	1.550 (3)	C14—H14A	0.9800
C2—C3	1.513 (3)	C15—C20	1.389 (3)
C2—C14	1.568 (3)	C15—C16	1.393 (3)
C3—C4	1.374 (3)	C16—C17	1.382 (3)
C3—C8	1.405 (3)	C16—H16A	0.9300
C4—C5	1.389 (3)	C17—C18	1.380 (3)
C4—H4A	0.9300	C17—H17A	0.9300
C5—C6	1.374 (3)	C18—C19	1.384 (3)
C6—C7	1.386 (3)	C18—H18A	0.9300
C6—H6A	0.9300	C19—C20	1.385 (3)
C7—C8	1.380 (3)	C19—H19A	0.9300
C7—H7A	0.9300	C20—H20A	0.9300
C9—C10	1.526 (3)		
			
C1—N1—C8	111.68 (19)	C9—C10—H10A	111.4
C1—N1—H1A	124.2	C11—C10—H10B	111.4
C8—N1—H1A	124.2	C9—C10—H10B	111.4
C2—N2—C9	120.73 (18)	H10A—C10—H10B	109.3
C2—N2—C12	109.45 (18)	C12—C11—C10	101.42 (19)
C9—N2—C12	108.40 (18)	C12—C11—H11A	111.5
O2—N3—O3	122.6 (2)	C10—C11—H11A	111.5
O2—N3—C5	118.6 (2)	C12—C11—H11B	111.5
O3—N3—C5	118.8 (2)	C10—C11—H11B	111.5
O4—N4—O5	123.8 (2)	H11A—C11—H11B	109.3
O4—N4—C13	117.0 (2)	N2—C12—C11	104.72 (19)
O5—N4—C13	119.1 (2)	N2—C12—C13	104.90 (18)
O1—C1—N1	126.6 (2)	C11—C12—C13	118.2 (2)
O1—C1—C2	125.2 (2)	N2—C12—H12A	109.5
N1—C1—C2	108.18 (19)	C11—C12—H12A	109.5
N2—C2—C3	120.44 (19)	C13—C12—H12A	109.5
N2—C2—C1	107.23 (17)	N4—C13—C14	113.83 (19)
C3—C2—C1	101.93 (18)	N4—C13—C12	110.60 (19)
N2—C2—C14	105.01 (17)	C14—C13—C12	104.84 (19)
C3—C2—C14	111.59 (18)	N4—C13—H13A	109.1
C1—C2—C14	110.43 (18)	C14—C13—H13A	109.1
C4—C3—C8	119.1 (2)	C12—C13—H13A	109.1
C4—C3—C2	132.6 (2)	C13—C14—C15	117.51 (19)
C8—C3—C2	108.11 (18)	C13—C14—C2	100.67 (17)
C3—C4—C5	117.6 (2)	C15—C14—C2	116.00 (18)
C3—C4—H4A	121.2	C13—C14—H14A	107.3
C5—C4—H4A	121.2	C15—C14—H14A	107.3
C6—C5—C4	123.5 (2)	C2—C14—H14A	107.3
C6—C5—N3	118.1 (2)	C20—C15—C16	117.6 (2)
C4—C5—N3	118.5 (2)	C20—C15—C14	119.3 (2)
C5—C6—C7	119.3 (2)	C16—C15—C14	123.1 (2)
C5—C6—H6A	120.3	C17—C16—C15	121.2 (2)
C7—C6—H6A	120.3	C17—C16—H16A	119.4
C8—C7—C6	117.7 (2)	C15—C16—H16A	119.4
C8—C7—H7A	121.1	C18—C17—C16	120.2 (2)
C6—C7—H7A	121.1	C18—C17—H17A	119.9
C7—C8—N1	127.4 (2)	C16—C17—H17A	119.9
C7—C8—C3	122.7 (2)	C17—C18—C19	119.8 (2)
N1—C8—C3	109.93 (19)	C17—C18—H18A	120.1
N2—C9—C10	104.50 (19)	C19—C18—H18A	120.1
N2—C9—H9A	110.9	C18—C19—C20	119.6 (2)
C10—C9—H9A	110.9	C18—C19—H19A	120.2
N2—C9—H9B	110.9	C20—C19—H19A	120.2
C10—C9—H9B	110.9	C19—C20—C15	121.6 (2)
H9A—C9—H9B	108.9	C19—C20—H20A	119.2
C11—C10—C9	101.72 (19)	C15—C20—H20A	119.2
C11—C10—H10A	111.4		
			
C8—N1—C1—O1	−178.1 (2)	C12—N2—C9—C10	13.3 (2)
C8—N1—C1—C2	1.6 (2)	N2—C9—C10—C11	−35.4 (2)
C9—N2—C2—C3	18.2 (3)	C9—C10—C11—C12	43.5 (2)
C12—N2—C2—C3	145.0 (2)	C2—N2—C12—C11	−119.4 (2)
C9—N2—C2—C1	133.9 (2)	C9—N2—C12—C11	14.2 (2)
C12—N2—C2—C1	−99.3 (2)	C2—N2—C12—C13	5.8 (2)
C9—N2—C2—C14	−108.6 (2)	C9—N2—C12—C13	139.27 (19)
C12—N2—C2—C14	18.2 (2)	C10—C11—C12—N2	−35.9 (2)
O1—C1—C2—N2	48.8 (3)	C10—C11—C12—C13	−152.1 (2)
N1—C1—C2—N2	−130.89 (19)	O4—N4—C13—C14	152.6 (2)
O1—C1—C2—C3	176.2 (2)	O5—N4—C13—C14	−29.7 (3)
N1—C1—C2—C3	−3.5 (2)	O4—N4—C13—C12	−89.7 (3)
O1—C1—C2—C14	−65.1 (3)	O5—N4—C13—C12	88.1 (3)
N1—C1—C2—C14	115.2 (2)	N2—C12—C13—N4	−151.47 (18)
N2—C2—C3—C4	−61.6 (3)	C11—C12—C13—N4	−35.3 (3)
C1—C2—C3—C4	180.0 (2)	N2—C12—C13—C14	−28.4 (2)
C14—C2—C3—C4	62.1 (3)	C11—C12—C13—C14	87.8 (2)
N2—C2—C3—C8	122.5 (2)	N4—C13—C14—C15	−73.9 (3)
C1—C2—C3—C8	4.1 (2)	C12—C13—C14—C15	165.08 (19)
C14—C2—C3—C8	−113.7 (2)	N4—C13—C14—C2	159.14 (19)
C8—C3—C4—C5	−1.1 (3)	C12—C13—C14—C2	38.1 (2)
C2—C3—C4—C5	−176.5 (2)	N2—C2—C14—C13	−34.7 (2)
C3—C4—C5—C6	3.1 (3)	C3—C2—C14—C13	−166.78 (18)
C3—C4—C5—N3	−176.6 (2)	C1—C2—C14—C13	80.6 (2)
O2—N3—C5—C6	−174.4 (2)	N2—C2—C14—C15	−162.62 (18)
O3—N3—C5—C6	5.0 (3)	C3—C2—C14—C15	65.3 (2)
O2—N3—C5—C4	5.3 (3)	C1—C2—C14—C15	−47.3 (2)
O3—N3—C5—C4	−175.2 (2)	C13—C14—C15—C20	144.2 (2)
C4—C5—C6—C7	−2.3 (4)	C2—C14—C15—C20	−96.7 (2)
N3—C5—C6—C7	177.4 (2)	C13—C14—C15—C16	−36.4 (3)
C5—C6—C7—C8	−0.5 (3)	C2—C14—C15—C16	82.7 (3)
C6—C7—C8—N1	−179.5 (2)	C20—C15—C16—C17	−0.6 (4)
C6—C7—C8—C3	2.5 (3)	C14—C15—C16—C17	179.9 (2)
C1—N1—C8—C7	−177.0 (2)	C15—C16—C17—C18	−0.2 (4)
C1—N1—C8—C3	1.2 (3)	C16—C17—C18—C19	1.2 (4)
C4—C3—C8—C7	−1.7 (3)	C17—C18—C19—C20	−1.4 (4)
C2—C3—C8—C7	174.8 (2)	C18—C19—C20—C15	0.6 (4)
C4—C3—C8—N1	180.0 (2)	C16—C15—C20—C19	0.4 (4)
C2—C3—C8—N1	−3.5 (2)	C14—C15—C20—C19	179.9 (2)
C2—N2—C9—C10	140.7 (2)		

### Hydrogen-bond geometry (Å, °)

**Table:**

*D*—H···*A*	*D*—H	H···*A*	*D*···*A*	*D*—H···*A*
N1—H1A···O1^i^	0.86	1.97	2.808 (2)	164

Symmetry codes: (i) −*x*+1, −*y*+2, −*z*+1.

## References

[bb1] Bruker (2007). *SMART* and *SAINT* Bruker AXS Inc., Madison, Wisconsin, USA.

[bb2] De March, P., Elias, L., Figueredo, M. & Font, J. (2002). *Tetrahedron*, **58**, 2667–2672.

[bb3] Fejes, I., Nyerges, M., Szollosy, A., Blasko, G. & Toke, L. (2001). *Tetrahedron*, **57**, 1129–1137.

[bb4] Karthikeyan, K., Perumal, P. T., Etti, S. & Shanmugam, G. (2007). *Tetrahedron*, **63**, 10581–10586.

[bb5] Liddell, J. R. (1998). *Nat. Prod. Rep.* **15**, 363–370. 10.1039/a815363y 9736995

[bb6] Michael, J. P. (1997). *Nat. Prod. Rep.* **14**, 619–636. 10.1039/np9971400619 9418297

[bb7] Sheldrick, G. M. (2008). *Acta Cryst.* A**64**, 112–122. 10.1107/S0108767307043930 18156677

[bb8] Usha, G., Selvanayagam, S., Velmurugan, D., Ravikumar, K. & Poornachandran, M. (2005*b*). *Acta Cryst.* E**61**, o3312–o3314.

[bb9] Usha, G., Selvanayagam, S., Velmurugan, D., Ravikumar, K. & Raghunathan, R. (2005*a*). *Acta Cryst.* E**61**, o3299–o3301.

